# Sustainable application of edible solute to control reservoir evaporation loss

**DOI:** 10.1038/s41598-025-28224-x

**Published:** 2025-12-24

**Authors:** Indramani Dhada, Poonam, Sudhakar Singha, Anoop Kumar Shukla

**Affiliations:** 1 Department of Civil Engineering, Indian Institute of Technology, Ropar, Rupnagar, Punjab 140001 India; 2 Department of Civil Engineering, GITAM (Deemed to be University), Hyderabad, Telangana 502329 India; 3 Manipal School of Architecture and Planning, Manipal Academy of Higher Education, Manipal, Karnataka 576104 India

**Keywords:** Evaporation, Reservoir management, Edible solutes, BEP analysis, Water conservation, Environmental sciences, Hydrology, Engineering

## Abstract

Recently, water preservation globally, particularly in Indian cities, has been prominently featured in newspaper headlines, underscoring its importance. This research explores the innovative use of edible solutes to tackle the challenges of evaporation in reservoirs, including water loss, increased salinity, and ecological disruptions. Traditional methods for controlling evaporation often have environmental drawbacks and high operational costs. By evaluating the environmental impact, cost-effectiveness, and feasibility of edible solutes such as mustard oil, neem oil, til oil, castor oil, cetyl alcohol, and stearyl alcohol, this study investigated their sustainable application in eight reservoirs across Andhra Pradesh and Telangana states in India. Through break-even analysis, the economic viability of edible solutes is compared to that of conventional methods over the lifespan of a reservoir. These findings suggest that edible solutes offer a promising and environmentally friendly alternative, reducing evaporation rates while minimizing the adverse effects on water quality and ecosystems. Despite the initial investment costs, the long-term savings and environmental benefits surpass those of the conventional approaches. This study estimated evaporation rates for eight reservoirs across Andhra Pradesh and Telangana in India (3049 mcm of water/year), showing a significant reduction when cetyl alcohol was used as a solute. Cetyl and stearyl alcohols are highlighted as practical and cost-effective evaporation retardants. Considering the cost of water at one *paise* per five litres of saved water, the break-even point (BEP) analysis for the adopted scenarios reveals that BEP is achieved for 30%, 10%, and 5% reduction in evaporation within one, two, and three months, respectively. Similarly, for scenario II (one paisa per one litre of saved water), the BEP was achieved at the beginning, 1.5 months, and 2.5 months, considering evaporation reduction by 30%, 10%, and 5%, respectively. Future research should validate the efficacy of microfilms in mitigating evaporation using time-resolved interferometry techniques. This study advocates sustainable water management practices and provides valuable insights for policymakers, water resource managers, and stakeholders seeking efficient solutions for evaporation control in reservoirs.

**Supplementary Information:**

The online version contains supplementary material available at 10.1038/s41598-025-28224-x.

## Introduction

The volume of evaporation loss from reservoirs, such as the high dam reservoir, significantly impacts water supply management by causing substantial annual capacity reductions, affecting water availability for various uses^1,2^. The volume of evaporation loss from reservoirs significantly impacts water supply management globally. Studies show that around 339.8 km^3^ of water evaporates annually from large reservoirs, nearly 73% of municipal water withdrawal in 2010, with a notable increase in evaporation volume over the years, especially in middle-income countries^3^. Additionally, small reservoirs in southern Europe have seen an 18.5% increase in cumulative area from 2000 to 2020, with evaporative losses potentially exceeding 400 Mm^3^ during warm months, affecting regions with chronic water stress issues^4^. Furthermore, projections for major reservoirs in the Contiguous United States indicate a potential increase in evaporation loss by 2.5 × 10^7^ m^3^/year (from 1980 to 2059), exacerbating water shortages, particularly in the southwestern US during summer/fall seasons^5^.

Evaporation loss in India has been a noteworthy concern, with studies showing varying trends over the years. Research indicates that evaporation has encouragingly decreased across the country, especially in regions like the North, Southwest, and Southeast, while the Northeast shows an increasing trend^6^. A detailed study^7^on the Bhakra and Pong reservoirs showed significant underestimation in evaporation losses up to 11.2% at Pong when linear height–area–storage (H–A–S) relationships were applied. This further led to errors in estimating the reservoir capacity, emphasizing the need to adopt nonlinear or piecewise-linear H–A–S models in reservoir planning for greater accuracy. These findings underline the importance of precise area estimations for accurate water balance modelling and storage sizing. Furthermore, published literature on Aji Reservoir in India^8^ reveals that utilizing surface covering by a monomolecular film (long-chain fatty alcohols, such as Cetyl and stearyl alcohols, which form a hydrophobic barrier over water surfaces) has shown promising results in reducing evaporation loss from open water surfaces, with savings ranging from 20% to 50% observed in various trials. Review on monomolecular films confirms evaporation reduction up to 40% is possible under optimal conditions using long-chain alcohols such as hexadecanol and octadecanol^9^. However, external factors like wind, dust, and microbial degradation are sensitive to performance. These findings underscore the importance of understanding and mitigating evaporation losses to ensure efficient water resource management practices in India and globally.

The accurate prediction of reservoir evaporation volume is crucial for successful dam management, aiding in efficient water supply planning and resource management to mitigate losses^10^. Various methods have been developed to reduce evaporation in different fields. Physical methods like floating or suspended covers can save significant amounts of water, ranging from 70% to 95%^11^. Additionally, a study by Youssef and Khodzinskaya (2019)^11^ shows that the use of thermal mixing by compressed air bubble plumes in deep reservoirs (> 18 m deep) through destratification is effective in suppressing evaporation, up to 15% in summer months. Chemical approaches, such as using products like Water Saver, can save between 20% and 40% of water but are widely utilized for evaporation reduction^12^. Biological methods like floating plants and wind breakers also promise to decrease evaporation volumes^3^. Furthermore, innovative solutions like low-cost polymer multilayer films have been proposed to reduce water evaporation significantly, with experiments showing a 29% reduction in cumulative evaporation under these films^13^. These diverse methods offer practical strategies for mitigating evaporation losses in various applications.

With the above background, although the neem, til and castor oils have been used at a large scale for their anti-oxidant properties, the effect of these oils over evaporation has not been studied earlier. Further, the economic feasibility of these solutes needs to be assessed through break-even point (BEP) analysis for real field application and to get a glimpse of the application process. This current research mainly focused on the following key objectives.

- To examine the innovative use of edible and biodegradable solutes to diminish evaporation, including loss of water, increase in salinity, and disruption to the ecosystem.
- To assess the environmental impact, cost-effectiveness, and practicality of edible solutes (mustard oil, neem oil, til oil, castor oil, cetyl alcohol, and stearyl alcohol) for evaporation reduction.
- To conduct an experimental study through the pan evaporation technique and validate it with existing analytical approaches.
- To conduct a break-even analysis comparing the economic viability of edible solutes against practices used conventionally, over the time of reservoir existence, considering eight reservoirs covering Andhra Pradesh and Telangana states in India.

The output of the study will be helpful to provide insights for policy makers, water resource managers, and stakeholders looking for sustainable water management and effective solutions for evaporation mitigation in reservoirs in light of increased water scarcity and impacts of climate change.

## Study area

**Fig. 1:**
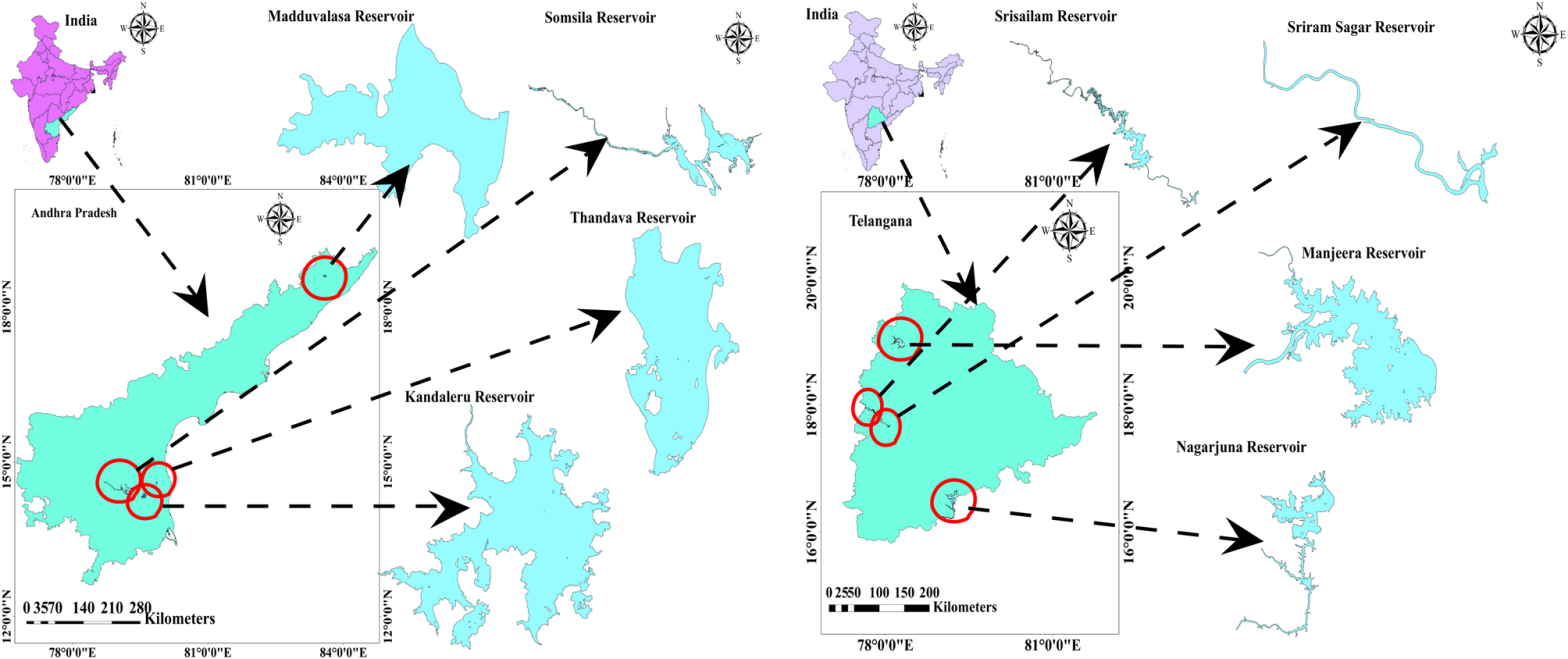
Geographical location of the study area.

The study encompasses four primary reservoirs, each across Telangana and Andhra Pradesh states, spanning the geographical expanse of India (Fig. 1). Four reservoirs, Kandaleru, Madduvalasa, Somasila, and Thandava, have been considered in the state of Andhra Pradesh, India (Fig. 1). The average water surface area for the Somasila reservoir was observed to be maximum, while that for the Madduvalasa reservoir was minimum. Moreover, for Telangana State, analysis extended to Manjeera, Nagarjuna, Sriram Sagar and Srisailam reservoirs, where Srisailam reservoir shows the most significant average water surface area, while Manjeera reservoir displayed the smallest (Table 1). Monthly water surface area estimates for each reservoir were derived from images extracted from the United States Geological Survey (USGS) and processed using ArcGIS software.

**Table 1:** Details of the reservoir selected for the study.

State	Name of the reservoir	River	District	Latitude	Longitude	Average water surface area (km^2^)
Andhra Pradesh	Kandaleru	Kandaleru	Nellore	14° 20’ 50.64"N	79° 35’38.76"E	36
	Madduvalasa	Suvarnamukhi	Srikakulam	18° 36’ 34.2"N	83° 35’12.84"E	14
	Somasila	Penna	Nellore	14° 29’ 27.24"N	79° 12’40.68"E	185
	Thandava	Thandava	East Godavari	17° 38’ 56.04"N	82° 27’ 32.4"E	23
Telangana	Manjeera	Manjeera	Manjeera	17°39’34.2"N	78° 3’ 37.08"E	5
	Nagarjuna	Krishna	Nalgonda and Palnadu	16° 33’ 0"N	79° 13’ 48"E	212
	Sriram Sagar	Godavari	Nizamabad	18° 58’ 6.96"N	78° 20’ 27.96"E	244
	Srisailam	Krishna	Nagarkurnool, Nandyal, Domalapenta	16° 5’ 12.84"N	78° 53’ 47.4"E	311

## Methodology

This research examines four reservoirs across Telangana State, employing experimental and empirical methods to assess evaporation rates. The experimental method utilizes the Pan Evaporimeter technique, while the empirical method encompasses four established formulas: the Penman equation, Meyer’s equation, Rohwer’s equation, and the Blaney-Criddle formula. The primary rationale for utilising these formulas stemmed from the availability of all necessary parameters through the NASA Power data access viewer and the objective to minimise error by deriving the average evaporation rate estimated from all methods. To pinpoint the location of the reservoir, the NASA Power data access viewer allowed for either inputting the latitude and longitude coordinates or searching directly on the map. Subsequently, monthly data were selected, focusing on the required parameters. The output was obtained in CSV format, facilitating the calculation of the average values of these factors to estimate the evaporation rate. Given the temperature fluctuation throughout the day, average values were considered to capture the net effect.

### Empirical method

#### Evaporation estimation using Penman formulae

The Penman equation is generally used to estimate evaporation from an open water surface, which was published by Howard Penman in 1948^14^. Equation 1: Penman’s equation needs daily air temperature, wind speed, atmospheric pressure, and radiation to predict evaporation. It is one of the accurate methods for evaporation estimation.

1 $$\:{E}_{o}=\frac{\left(700{T}_{M}\:\right)/\left(100-A\right)+15\left(T-{T}_{d}\right)}{\left(80-T\right)}\left(mm/day\right)$$

where *E*_*o*_ represents the rate of evaporation in mm/day per unit surface area ℃, *T*_*m*_ = *T + 0.006 h*, *h* represents water surface area elevation with respect to mean sea level in (m), *T* = desired month average atmospheric temperature, *A* represents the latitude (degree), and *T*_*d*_ = desired month atmospheric average dew point.

#### Evaporation estimation using Meyer’s formula

Meyer’s formula helps estimate evaporation losses in diverse climates^15^.

2 $$\:{E}_{L}={K}_{M}\left({e}_{w-}{e}_{a}\right)\left[1+\frac{{u}_{9}}{16}\right]$$

where, *E*_*L*_ represents unit surface area wise lake evaporation (mm/day), *K*_*M*_ = coefficient considered due to various factors (*K*_*M*_=0.36 for large deep water and *K*_*M*_=0.50 for small shallow lakes), *e*_*w*_ represents water surface saturation vapour pressure (mm of mercury), *e*_*a*_ is specific height actual vapor pressure of the overlying air (mm of mercury), *u*_*9*_ is monthly average wind velocity in kmph at 9 m height above the ground. The wind velocity extracted from NASA POWER data access is m/s at 10 m height. Therefore, a conversion from U_9_ to U_10_ was necessary, employing the 1/7 power law.

#### Evaporation estimation using Rohwer’s formulae

Rohwer`s formula^16^ has derived an expression to estimate the evaporation, which is given in Eq. 3. Accounts for the effect of pressure in addition to the wind speed effect.

3 $$\:{E}_{L}=0.771(1.465-0.000732{p}_{a})(0.44+0.0733{u}_{o}{\left)\right(e}_{w}{-e}_{a})$$

where, *p*_*a*_ =mean barometric pressure (mm of mercury), *u*_*o*_ = mean wind velocity in kmph at 0.6 m height above the ground, *E*_*L*_=lake evaporation (mm/day) per unit surface area, *e*_*w*_ =saturation vapour pressure at the water surface temperature (mm of mercury), *e*_*a*_ =actual vapor pressure of the overlying air at a specified height (mm of mercury). The wind velocity can be assumed to follow the 1/7 power law.

4 $${\mathrm{U}}_{\rm h} = {\mathrm{Ch}}^{1/7}$$

where, U_h_ = wind velocity at a height h above the ground and C = constant. This equation can be used to determine the velocity at any desired level.

Rohwer’s equation requires temperature, barometric pressure, relative humidity, and wind velocity at 0.6 m above ground level. Atmospheric pressure was obtained in kPa from the data access, and wind velocity was given in m/s at 10 m height. Conversion was performed to acquire atmospheric pressure in mm Hg and wind velocity at 0.6 m above ground level in km/h.

#### Evaporation estimation using Blaney-Criddle formulae

Blaney Criddle Equation has derived an expression to estimate the evaporation^17^, which is given in Eq. 5. This equation considers temperature, daylight hours, and annual daylight hours.

5 $$\:{E}_{L}=\left(0.0173{T}_{a-}0.314\right){T}_{a}\frac{D}{{D}_{TA}}25.4$$

where, D = the daylight hours, D_TA_ is the total annual daylight hours, and T_a_ is the overlying air temperature in ℉. Similarly, the temperature was converted from degrees Celsius to Fahrenheit for the computation of E_L_.

The details of the parameters used in evaporation computation by different methods are given in Table 2. Further, the limitations of each method are given in Table S1.

**Table 2:** Parameters used for computing evaporation.

Name	Equations	K_M_	WS	RH	h	A	T_d_	p_a_	D	*D*_*TA*_	*e*_*w*_	*e*_*a*_
Pan evaporation	E_L_ = 0.7*pan evaporation	X	X	X	X	X	X	X	X	X	X	X
Penman equation	${E}_{o}=\frac{(700{T}_{M} )/\left(100-A\right)+15\left(T-{T}_{d}\right)}{\left(80-T\right)}\left(mm/day\right)$ T_M=_T + 0.006 h	✔	X	X	X	✔	✔	✔	X	X	X	X
Meyer’s formula	*E*_*L*_ = *K*_*M*_(*e*_*w*_ − *e*_*a*_) x [1 + *u*_9_/16]	✔	✔	✔	✔	X	X	X	X	X	X	✔
Rohwer`s Formula	*E*_*L*_ = 0.771 x (1.465 − 0.000732p_a_) x (0.44 + 0.0733*u*_o_) x(*e*_*w*_ − *e*_*a*_)	✔	X	✔	✔	X	X	X	✔	X	X	✔
Blaney Criddle Equation	${E}_{L}=\left(0.0173{T}_{a-}0.314\right) {T}_{a}\frac{D}{{D}_{TA}}25.4$	✔	X	X	X	X	X	X	X	✔	✔	X

E_L_ or E_o_ = Lake evaporation, WS = Wind Speed, RH = Relative Humidity, h = Elevation of water surface area with respect to mean sea level in (m), A = Latitude, T_d_ = Mean dew point of the atmosphere, e_w_ = Saturation vapor pressure, e_a_ = Actual vapor pressure, p_a_ = Mean barometric pressure, D = Hours of daylight, D_TA_ = Total annual daylight hours, K_M_ =Coefficient, u_9_ = mean wind speed at 9 m height from ground (Km/h), T = Monthly average atmospheric temperature.

#### Experimental method

**Fig. 2:**
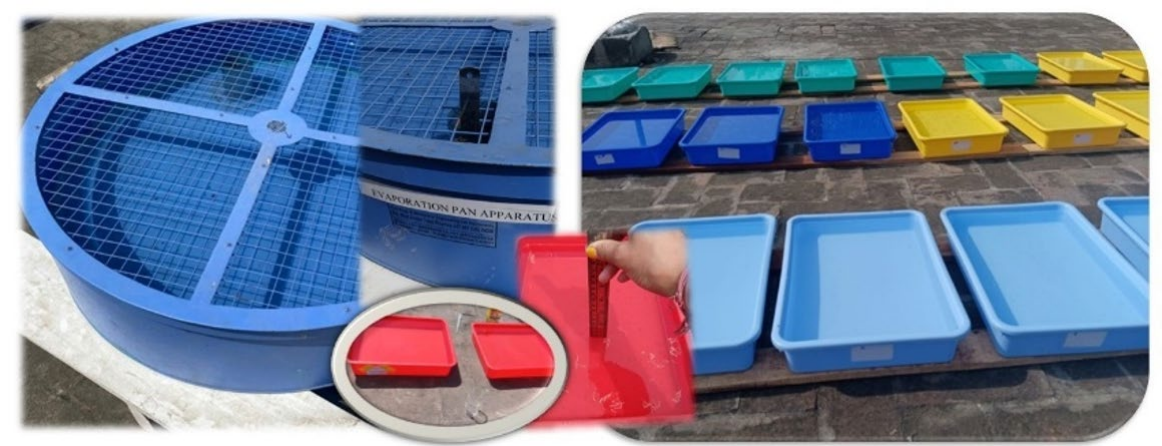
Standard evaporation pans and trays.

The present research employs the Pan Evaporation method as an experimental approach. However, various evaporimeters such as the Class-A evaporation pan, ISI standard pan, Colorado Sunken pan, and US Geological Survey floating pan are available globally. Among them, the Class-A Evaporation Pan stands out as the most commonly utilized for field experiments. In this study, a Class-A, also known as Class A Land Pan (as prescribed in IS 5973:1970), a circular container having a 121 cm diameter and 25.5 cm depth, was used to measure daily evaporation. To allow the air to circulate freely and avoid temperature transmission from the ground to the bottom of the pan, the pan was placed on top of a 15 cm high wooden frame. Evaporation was measured by measuring the depth of water in the pan with a gauge hook. Considering some natural losses, the water level is maintained at around 5 cm below the rim of the pan. The water level was measured for over 24 h on a daily basis during the study. In the present study, a total of 81 rectangular pans/trays measuring 38 × 27 cm with a depth of 7 cm were utilized to investigate the quantity of evaporated water applying various chemical/solute films. The use of this standard pan ensures comparability with past studies and aligns with national guidelines^18^. Furthermore, the ratio between evaporation rates in the rectangular pans and those in the Class-A pan was considered to estimate the actual evaporation relative to the Class-A pan. As the Class-A evaporation pan was not an exact model of any large lake or reservoir, the observed evaporation from the pan had to be corrected to obtain the evaporation from the lake under the same or similar climatic conditions. Thus, the corrected evaporation from the lake was estimated by considering a pan coefficient. The pan coefficient (C_p_) for Class-A evaporation pan varies in the range of 0.6–0.8, and here, in the present study, it has been considered as the average value of 0.7. On the first day, doses of 10 mg, 20 mg, 30 mg, and 40 mg of Cetyl and Stearyl alcohol were evenly distributed in the pan/tray, each in triplicate, and observed for evaporation over 5 days. Similarly, Castor oil, Neem oil, Mustard oil, and Til oil were employed at doses of 2 ml, 4 ml, 6 ml, and 8 ml, respectively (in triplicate, Fig. 2). Meteorological parameters such as temperature, relative humidity, and wind speed were recorded using an Anemometer (Model - AVM-06, HTC instruments) to compare the experimentally observed evaporation rates with those derived from empirical methods.

### Estimation of water volume

**Fig. 3:**
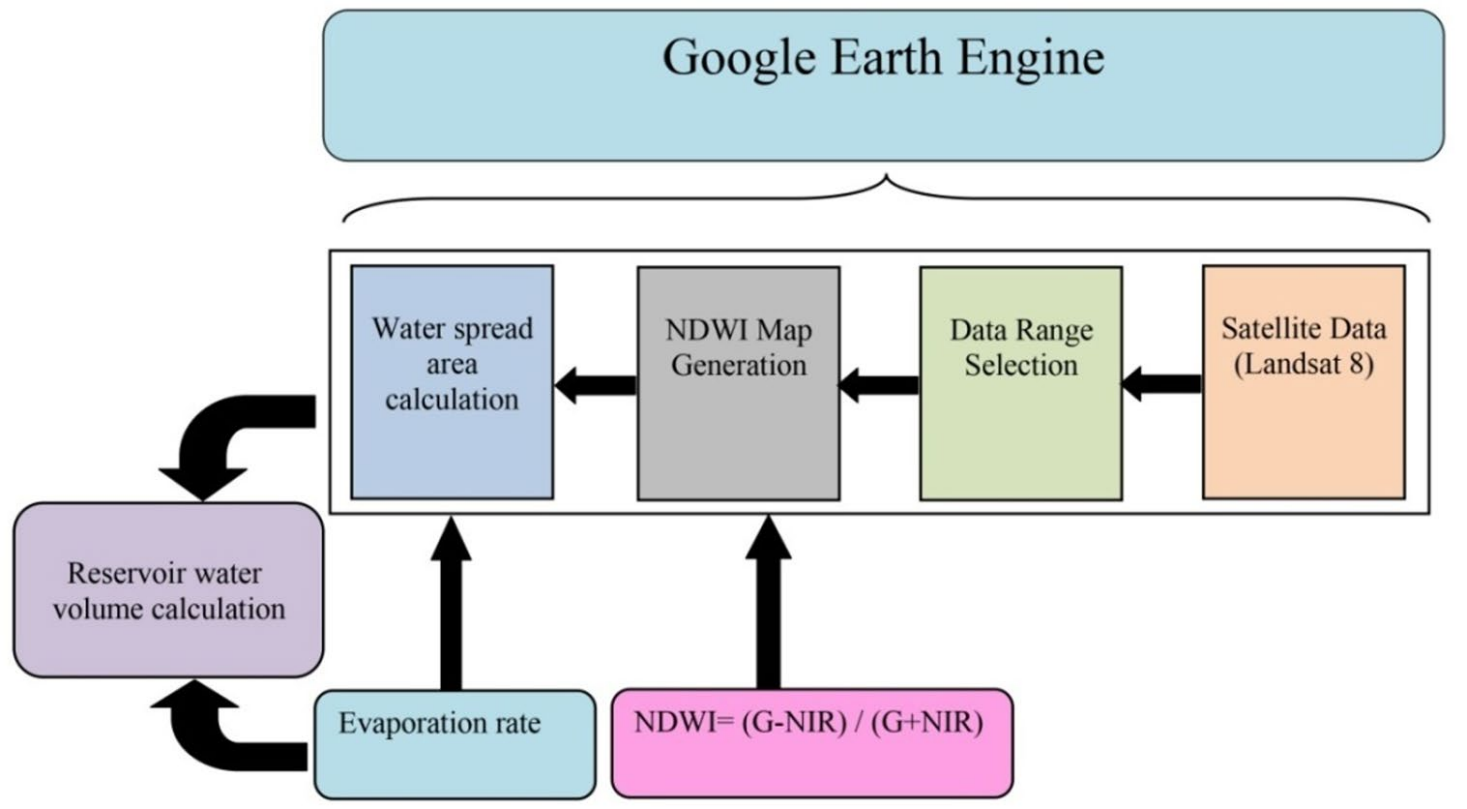
Steps to estimate water volume in the reservoir.

The decrease in the reservoir surface area of the corresponding water levels can be used to illustrate the decline in reservoir capacity. The current research extracted water pixels using the Normalized Difference Water Index (NDWI). The NDWI approach distinguishes between open water features, enhances their visibility in remotely sensed digital imaging, and simultaneously removes features of soil and terrestrial plants. The formula to estimate NDWI (equation-6) is given as:

6 $$NDWI = (G-NIR) / (G + NIR)$$

where G is the spectral reflectance in the green band, NIR is the near-infrared band of the digital image considered. Figure 3 depicts the water volume estimation steps adopted in the current study.

### Method to control evaporation loss

Evaporation losses can be minimized by three popular methods: physical, biological and chemical. In the present study, the chemical method is being adopted to investigate the reduction of evaporation loss from the selected reservoirs.

#### Chemical method

The chemical water evaporation retardants have been used to monitor the evaporation. Chemical methods offer several advantages in reducing evaporation. They have been proven effective in decreasing evaporation rates from water surfaces, with some methods saving water between 20% to 95%^11^. Chemical films containing Cetyl and stearyl alcohols have successfully reduced evaporation from open water surfaces, increasing storage efficiency^19^. It has been found that chemicals that can form a thin monomolecular coating can reduce water surface evaporation loss. Energy inputs from the atmosphere are reflected due to the film’s creation, lowering evaporation loss. Aquatic life is unharmed by the film because it allows enough air to pass through it. The most successful film for regulating evaporation is one formed from different grades of fatty alcohols. When these chemicals are applied to the water’s surface, they create a mono-molecular layer, that serves as a wall between the water and the air. Fatty alcohols are utilized as chemical water evaporation retardants (WERs) and can be found in powder, solution, or emulsion form. These chemical WERs have a high application cost, which is a drawback. However, the amount of water conserved by this method would be less expensive in times of scarcity, such as drought, than alternative means of delivering water from remote regions via human or mechanical delivery. The economics of WER utilization may fluctuate from site to site depending on regional factors. Chemical WERs also have the problem of monolayer breakup at high wind speeds. In the present study, the following substances were employed to reduce the water evaporation: Cetyl Alcohol (Hexadecanol) C_16_H_33_OH, Stearyl Alcohol (Octadecanol) C_18_H_37_OH. The details of Cetyl alcohol and Stearyl alcohol properties are provided in Table 3.

**Table 3:** Properties of cetyl alcohol stearyl alcohol.

Chemical formula	Cetyl alcohol (C_16_H_34_O)	Stearyl alcohol (C_18_H_38_O)
Molar mass	242.447 g.mol^-1^	270.49 g.mol^-1^
Appearance	White crystal or flakes	White crystal or flakes
Odor	Very faint, waxy	-
Density	0.811 g/cm^3^	0.812 g/cm^3^
Melting point	49.3 °C	59.4 to 59.8 °C
Boiling point	344 °C	210 °C at 15 mm Hg (2.0 kPa)
Solubility in water	Insoluble	1.1 × 10-3 mg/l
solubility	Very soluble in ether, benzene, and chloroform, soluble in acetone, and slightly soluble in alcohol.	-

#### Physical method (Spreading the materials over the water surface)

The WER material can be applied to the open sea surface using three techniques. Both flakes and lumps of Cetyl /Stearyl alcohol are available, with a melting point of approximately 49 °C. The substance is pulverized using a cold method to prepare it for spreading. For even distribution across the water’s surface, the underlying material should be between 60 and 100 mesh, and application is facilitated using a motorized boat and a powder duster. Ensuring uniform application across the water’s surface is essential. Kerosene or turpentine was used to dissolve Cetyl /Stearyl alcohol, which was then applied to the water’s surface. A one-molecule-thick lipid film was created at the air/water interface because the solution spread quickly and the solvent evaporated. Due to their lower volatility, kerosene and turpentine shouldn’t be chosen because of the potential for long-lasting odours. Ether can be employed since it quickly evaporates after application, leaving a monomolecular layer of Cetyl/Stearyl alcohol on the water’s surface. In this instance, the cost of the solvent increases the cost of spreading. In some nations, spreading the substance as an emulsion on the water’s surface is preferred, but the outcome of this more straightforward and less expensive method is still uncertain. By boat or stationary rafts with an arrangement to feed emulsion on the water’s surface drop by drop, the emulsion can be applied to the water’s surface.

In this study, different oils: Castor oil, Til oil, Mustard oil, and Neem oil, Cetyl and Stearyl alcohol, are used to reduce evaporation, where Cetyl and Stearyl alcohol are spread in powder form. The details of castor oil, til oil, mustard oil, and neem oil properties have been provided in Table 4.

**Table 4:** Physical properties of castor oil, Neem oil, Til oil, mustard oil.

Solute Properties	Castor oil	Neem oil	Til oil	Mustard oil
Density	0.967gm/ml at 25 °C	0.84 g/ml	0.921–0.924 g/cm^3^ at 15 °C	0.91 g/cm^3^
Solubility in water	Very poor	Emulsifies with water	Insoluble	2 g/L (20 °C)
Melting point	-10 to -18 °C	14 °C	-6 to -3 °C	-80 °C
Flashpoint (°C)	229 °C	More than 170 °C – no fire Hazard	255 °C	36.3 °C
Boiling point	313 °C	> 100 °C	338–366 °C	151.9 °C at 760 mm Hg
odor	Faint, mild odour	Brown Viscous liquid	Almost odourless	-
Viscosity	6–8 poise at 25 °C	Viscous material	65 mPa-s at 20 °C	-
Refractive Index	1.473–1.477 at 25 °C	-	1.473–1.476 at 20 °C	1.484
Vapour Pressure	-	38 to 200 °C	9.0*10^− 5^ mm-Hg at 25 °C	4.57 mm Hg at 25 °C

### Break-even point (BEP) analysis

As demonstrated in various studies, break-even point (BEP) analysis can be utilized to forecast future profitability^20,21^. BEP is a point where the total cost (expenditure) equals the total revenue generated. It determines the number of years or the amount of revenue that`s needed to cover the total cost. The total cost includes fixed costs (including motor boat, equipment cost, property taxes, etc.) and variable costs (including raw materials, utilities, chemicals, labour, spreading activity, and hidden costs). At the BEP, the total cost of chemicals used, labour, machinery, and the total water saved equals, meaning there is no loss or gain. Two cases have been considered; the cost of water is assumed to be one *paise* per five liters of water saved and one *paise* per liter in estimating BEP.

## Results

**Fig. 4:**
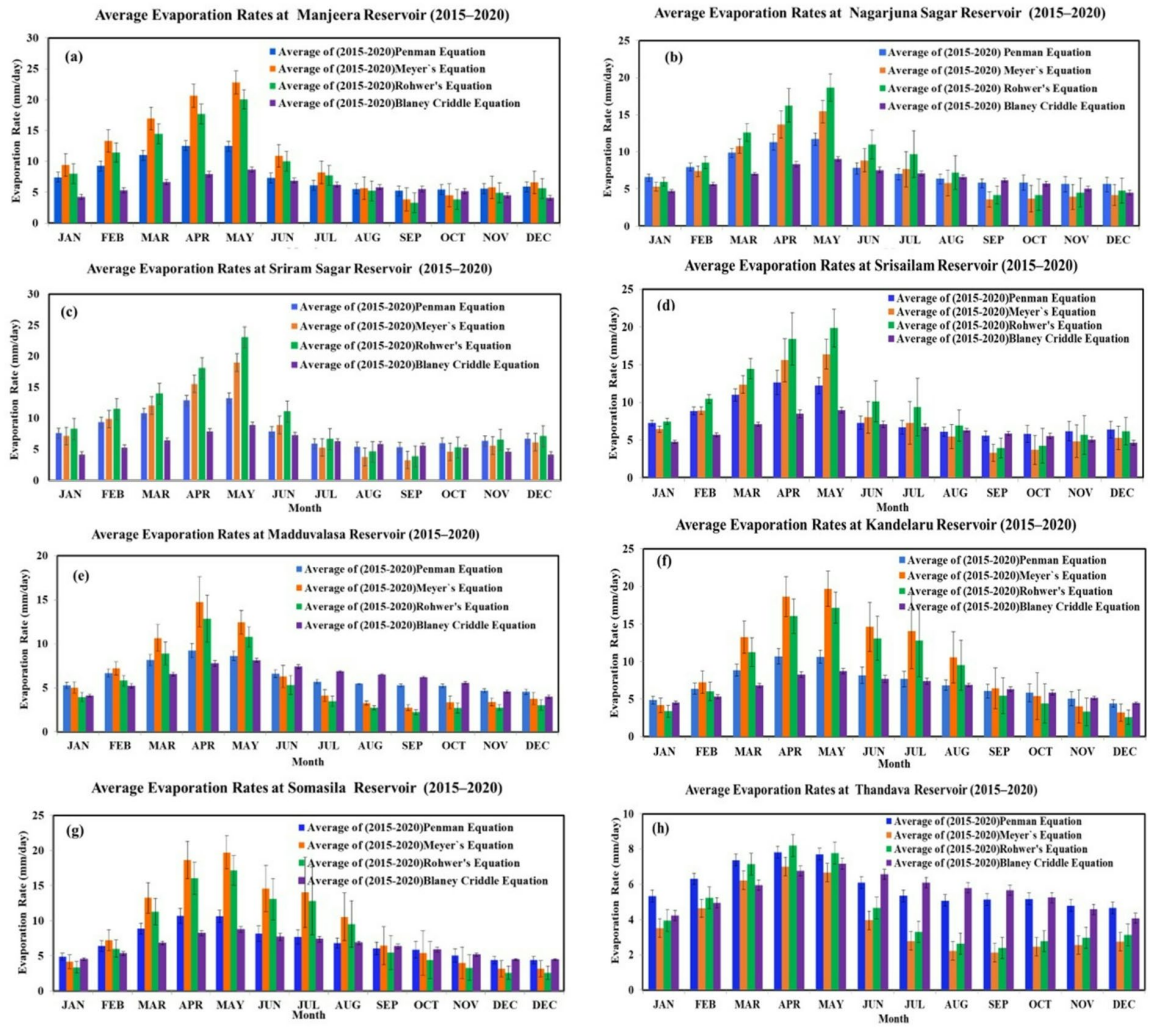
Average evaporation rate of (a) Manjeera, (b) Nagarjuna Sagar, (c) Sriram, (d) Srisailam reservoir, (e) Madduvalasa, (f) Kandaleru, (g) Somasila, and (h) Thandava.

The variations of evaporation rates for all the reservoirs have been observed, and a comparison analysis was carried out. The season-wise details of the evaporation capacity/rate of respective reservoirs have been presented in Fig. 4 (a to h), where it can be noted that Kandaleru (14.06 ± 1.41 mm/day) and Somasila (14.06 ± 0.95 mm/day) reservoirs of Andhra Pradesh contributed the highest evaporation rates during hot summer (May), followed by Madduvalasa (11.16 ± 1.28 mm/day) and Thandava (8.32 ± 0.84 mm/day). In Telangana, Manjeera (16.08 ± 1.33 mm/day) and Sriram (16.04 ± 5.32 mm/day) reservoirs contributed to the highest evaporation rates in the hot summer (May), followed by Srisailam (14.36 ± 1.48 mm/day) and Nagarjuna (13.70 ± 1.13 mm/day) reservoirs. In comparison to summer, the evaporation rates were recorded with lower values in winter (November), where evaporation rates followed the sequence as Madduvalasa (3.84 ± 0.22 mm/day), Kandaleru (3.68 ± 0.69 mm/day), Somasila (3.68 ± 0.45 mm/day), and Thandava (3.65 ± 0.48 mm/day) in Andhra Pradesh state. Reservoirs from Telangana, such as Nagarjuna Sagar (4.78 ± 1.11 mm/day), contributed the highest evaporation rate, followed by Srisailam (4.65 ± 0.84 mm/day), Manjeera (4.53 ± 0.66 mm/day), and Sriram (4.49 ± 0.91 mm/day) (Fig. 4a–h).

### Impact of oil and chemicals on the water surface

**Fig. 5:**
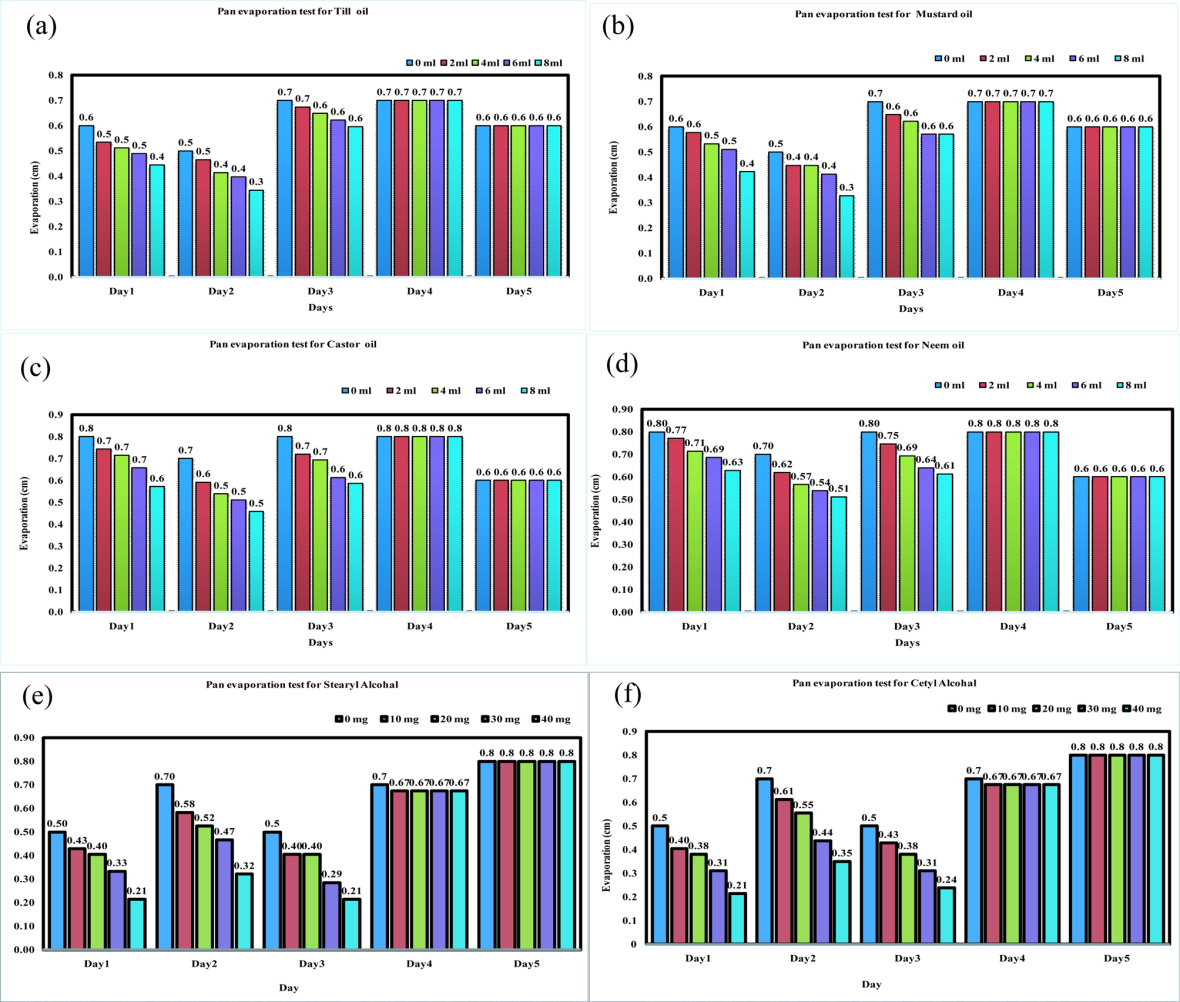
Variation of evaporation rate with varying dose and time for (a) Til oil, (b) Mustard oil, (c) Castor oil, (d) Neem oil, (e) Stearyl alcohol and (f) Cetyl alcohol.

The observation of evaporation loss variation due to the application of til oil, mustard oil, neem oil, castor oil, stearyl alcohol, and cetyl alcohol was carried out for five days with different dose of oils (0 ml, 2 ml, 4 ml, 6 ml, and 8 ml) and alcohols (0 mg, 10 mg, 20 mg, 30 mg, and 40 mg). Figure 5 shows the decrease in the evaporation rate with an increase in the dose of edible solutes considered for the present study.

Moreover, it has been observed that the life of the applied film of all the solutes lasts for three days and, after that, vanishes, which highlights that the efficiency of the chemical film tends to reduce to zero. On the fourth day, the percentage reduction of evaporation was the same as that of the water without any solutes, which directly implies the absence of a solute layer on the water’s surface. A gradually declining linear relation was observed between different solute doses and the evaporation loss for all the solutes used in the present study, further replicating that a higher dose of solutes contributes to a higher reduction in evaporation loss (Fig. 5).

### Cost analysis of edible solutes

The cost comparison analysis was carried out between all the oils and alcohols separately, where it can be noted that Castor oil is the most economical solute among the oils used to reduce evaporation as it has a very low cost of about Rs. 165 per kg and the quantity of dose required to reduce evaporation up to a specific limit is minimum (Table 5). The cost was considered as per the market price during the present study. The experimental results showed that a minimum dose of Castor oil (8.6 ml) was required among all other oils to reduce the evaporation rate of water up to 30 per cent. Cetyl alcohol was observed as the most economical and beneficial chemical for reducing evaporation from lakes and reservoirs, as it has a very low cost. Further, cetyl alcohol does not harm aquatic life, allows sunlight to penetrate the water, and allows re-oxygenation, but does not allow water to evaporate easily.

**Table 5:** Cost and dose comparison of different solutes.

Name of Solutes	Solute required for 30% evaporation reduction in the standard pan (in ml or mg)	Solute required for 30% evaporation reduction in standard pan (in ml/m^2^ or mg/m^2^ surface area)	Price of solute based on dose to reduce 30% evaporation (in rupees)	Price of solute per kg (or 1000 ml) in rupees	Reference
Til oil	11.38 ml	9.90 ml	3.30	290	Vihan Herbal and Food Ingredients Bhopal 2022
Mustard oil	9.9 ml	8.61 ml	1.90	192	Fortune Mustard Oils 2020^22^
Castor oil	8.6 ml	7.48 ml	1.42	165	Shiv Sales Corporation
Neem oil	10.69 ml	9.30 ml	2.14	200	HNCO Organics Private Limited 2022^23^
Stearyl alcohol	30.86 mg	26.83 mg	0.03	1020	Oswal 2021
Cetyl alcohol	30.56 mg	26.57 mg	0.03	1000	Oswal 2021

*Note: Standard Pan surface area 1.15m^2^.

### Break-even point (BEP) scenario

**Fig. 6:**
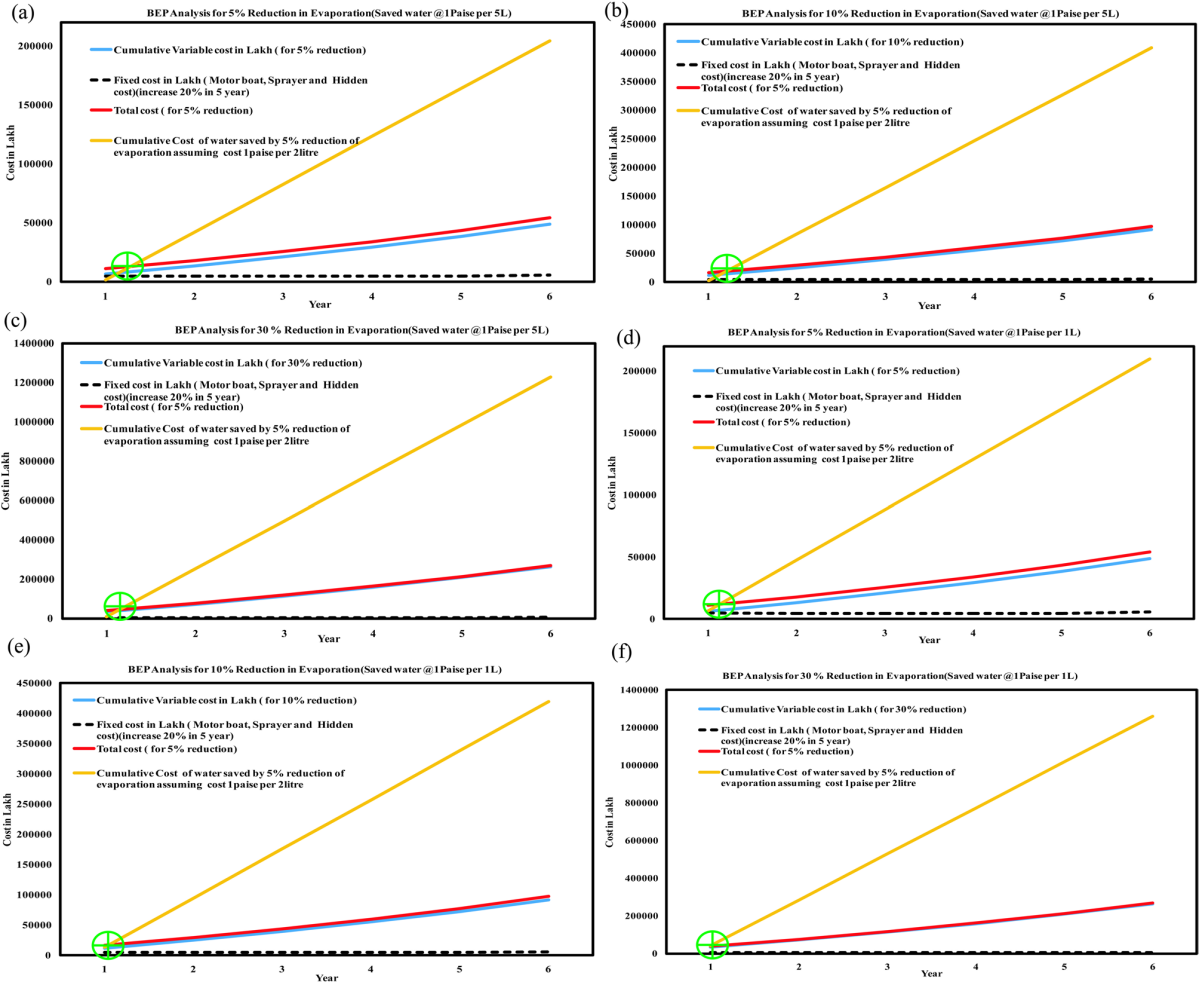
BEP for 5%, 10%, and 30% reduction of total evaporation considering cost of saved water @ one *paisa*/5L (a, b,c) and one *paisa*/L (d, e,f).

The BEP analysis has been undertaken by considering the actual cost of raw materials (boat, sprayer etc., increased @20% over a span of five years’ intervals), cost of labour (increased @ 10% in each successive year), cost of solute (increased @ 10% in each subsequent year), hidden cost (@ 20% over a span of five years’ interval and the cost of water saved (Fig. 6: scenario I @ one *paise* per five liters and Scenario II, @ one *paise* per liter of saved water).

The cost of solute and labour has been taken as per the present market cost, and the price of the boat and sprayer has been taken from the literature^24^ and the pilot study conducted by Jal Shakti Delhi and modified after considering other factors. The cost can be reduced if other mechanized means of sprayers are chosen, where the initial investment may increase, but the BEP time period can be lowered.

#### Scenario-I (Considering the cost of saved water @ one paisa/5L*)*

Figure 6 (a), (b), (c) shows the BEP analysis, where the x-axis represents the number of years and the y-axis indicates the cost in lakhs. In the present study, considering the price of saved water @ one *paisa*/five litres for reduction of 5%, 10%, and 30% of total evaporation (scenario I) has been performed. The result shows that in scenario I, the BEP was achieved after 3 months, 2 months, and 1 month, with a reduction of 5%,10%, and 30% of the total evaporation, respectively. The expenditure till achieving the BEP is Rs. 0.12 Lakhs and Rs. 0.2 Lakhs and Rs. 0.45 Lakhs for the case of 5%, 10%, and 30% reduction of total evaporation, respectively.

#### Scenario-II (Considering the cost of saved water @ one paisa/1L*)*

Similarly, Fig. 6 (d), (e), (f) shows the BEP analysis for scenario II, for the cost of saved water @ one *paisa*/L The result shows the BEP was achieved after 2.5 months, 1.5 months, and at the beginning (from the start, the technology implementation will assure a profit), with a reduction of 5%,10%, and 30% of total evaporation, respectively. The expenditure till achieving the BEP is Rs. 0.14 Lakhs, Rs. 0.18 Lakhs, and Rs. 0.50 Lakhs for the case of 5%, 10%, and 30% reduction of total evaporation, respectively. If we attempt a reduction exceeding 30%, the technology demonstrates benefits from the outset. Similarly, the BEP vs. achieved time (year) may be plotted for various percentages of reduction in evaporation to find the profit margin.

## Discussion

The findings of this study elucidate promising insights into mitigating water loss due to evaporation in eight reservoirs, particularly in India’s two drought states (Andhra Pradesh and Telangana). The observed evaporation of 1354 mcm per year (average evaporation rate of 2951 mm/year; Table 6) underscores the significance of addressing this issue, especially in regions where water resources are already under stress. Furthermore, identifying Cetyl and Stearyl alcohols as practical and cost-effective evaporation retardants highlights the potential for scalable solutions to combat water loss in reservoirs.

**Table 6:** Details of reservoirs with surface area and volume.

State	Name of Reservoir	Average water surface area (km^2^)	Evaporation rate ( mm/year)	The volume of evaporation water (considering average pan factor 0.7) (mcm)
Andhra Pradesh	Kandaleru	36	3360	51
	Madduvalasa	14	2246	13
	Somasila	185	3360	259
	Thandava	23	1930	18
Telangana	Nagarjuna	212	2978	264
	Manjeera	5	3323	6
	Sriram Sagar	244	3192	325
	Srisailam	311	3218	418

Cetyl and stearyl alcohols are known to degrade into non-toxic fatty acids and have limited toxicity^25,26^ However, long-term accumulation in sediments and chronic exposure impacts have not been thoroughly studied, emphasizing the need for ecotoxicological field assessments before large-scale implementation. Cetyl alcohol demonstrated significant short-term efficacy; repeated applications over long durations may lead to ecological changes in sediment or aquatic fauna^27^. Rapid biodegradability of Cetyl alcohol by naturally available microbes under ambient conditions is an advantage, which supports ecological balance by minimizing risk due to ecotoxic byproducts^28^, but further studies are needed to assess long-term safety.

From the literature, it is observed that there is an increase in shear imposed on the monolayer with an increase in wind speed^29^, and the relationship between the internal angle of the wedge and wind velocity follows a power law. Higher wind velocities (> 2.5 m/s) led to partial breakup of the monolayer films, reducing efficacy, whereas temperature variations (25–35 °C) had minimal impact within this range. In the present study, the temperature was observed in the range of 25–30 °C and relative humidity in the range of 19–24%. Although a monolayer can save 41% of the evaporation, in the absence of radiation, a 9 m/s wind caused ~ 15 mm/day evaporation. Wind speed from 0 to 9 m/s can deteriorate the effectiveness of the monolayer from 60 to 13% ^30^. The pan evaporation estimates with the triplicate samples in the present study were compared with the average evaporation derived from empirical methods, considering the weather parameters of the experimental method, which demonstrates similar trends (with a variation of a maximum 6%). Future work would focus on quantitative calibration and validation of empirical methods with a larger number of field-observed pan data for greater accuracy.

The t-test results in the evaporation reduction of various oils (Til oil, Mustard oil, Castor oil, and Neem oil) and alcohols (Cetyl and Steryl alcohol) compared to the control pan evaporation have been represented in Table 7. The t-statistics of Til oil (3.02), Mustard oil (2.92), Cetyl alcohol (4.21), and Steryl alcohol (4.13) were all larger than the critical t-values and as such are statistically significant as they are associated with p-values much less than 0.05 at a 95% confidence level (α = 0.05), thus the differences between these samples merit further exploration and indicate that they exhibit unique measurable characteristics in relation to Til oil, Mustard oil, and Castor oil. Castor oil (t = 0.55, *p* ≈ 0.30) and Neem oil (t = 0.009, *p* ≈ 0.99), however, demonstrate no statistical significance, as the parameters selected for testing (t) were well below the critical level. The probability (p-values) greater than 0.05 indicate that any measured deviations observed from classical control are likely a result of random error, rather than an explicit difference between the oils or alcohols. In conclusion, this study demonstrated that Til oil, Mustard oil, Cetyl alcohol, and Steryl alcohol exhibit evaporation characteristics, which are statistically different; in contrast, Castor and Neem oils exhibited no significant differences under the conditions assessed. Overall, the Cetyl alcohol is more important in reducing evaporation than the control pan evaporation. The experimental results demonstrating the efficacy of Cetyl alcohol, particularly at a dosage of 26.57 mg per m^2^, in significantly reducing evaporation rates are noteworthy. Findings of this research are consistent with the results published by Panjabi et al.^24^, who observed a 20–50% reduction in evaporation using monomolecular films in Indian reservoirs.

**Table 7:** Statistical significance of evaporation reduction by the solutes.

Comparing t-test two-sample	Til oil	Mustard oil	Castor oil	Neem Oil	Cetyl alcohol	Stearyl alcohol
T-Stat	3.021	2.919	0.551	0.009	4.210	4.132
Probability (T < = t) two-tailed	0.010	0.019	0.298	0.993	0.004	0.006
t Critical two-tailed	2.365	2.306	2.306	2.306	2.365	2.447

The cost-benefit analysis, as demonstrated by the BEP results, indicates a tangible economic benefit in adopting technologies that reduce evaporation by more than 30%. With the assumed price of saved water at one *paise* per five liters, the cost savings associated with implementing such measures become apparent. Moreover, the investigation into microfilm, specifically thin monomolecular layers, presents an intriguing avenue for further research. Overall, these findings underscore the potential of practical interventions, such as applying cetyl and stearyl alcohols or developing microfilms, to mitigate water loss through evaporation in reservoirs significantly. Notably, the demonstrated economic viability of such measures suggests that they could be feasibly implemented on a larger scale, offering tangible benefits for water conservation efforts in the region. However, further research and field testing will be essential to validate and optimize these approaches for real-world applications fully.

## Conclusions

The evaporation rate of water for eight reservoirs in two states of India was estimated. It was observed that 3246 mcm of water/year evaporated in these reservoirs. The experimental results demonstrated that evaporation was reduced significantly for cetyl alcohol (26.57 mg dose per m^2^ surface area, considering a 30% reduction in evaporation compared to plain water without solute) among all the solutes studied. Furthermore, it was determined that cetyl and stearyl alcohols are among the most practical and cost-effective evaporation retardants, significantly decreasing evaporation. The BEP results indicate a benefit from the outset if more than a 30% reduction of evaporation is considered for the price of saved water, taken to be @ one paise per five litres. The adopted method demonstrated significant benefits when applied in the field. The investigation was undertaken to verify the microfilm (thin monomolecular layer) that is imperceptible over the water’s surface and considerably decreases evaporation, which may be further elucidated through time-resolved interferometry techniques. The present study focused only on two states facing adverse climatic conditions regarding significant water scarcity: Andhra Pradesh and Telangana, comprising eight reservoirs. However, reservoirs from diverse climatic conditions could further enlighten the present outcome.

### Future scope

The study was conducted on eight reservoirs in Andhra Pradesh and Telangana State; however, future research could extend this analysis to all reservoirs across India. This investigation estimated evaporation using four empirical equations; nevertheless, a more comprehensive study could be undertaken employing additional methods considering diverse parameters correlated with evaporation rates. To reduce evaporation, experiments could be conducted using combinations of various solutes in different proportions to determine an optimal solute mixture, as it has been observed that the combination of cetyl and stearyl alcohols is most effective in terms of bonding with water due to their hydrophobic and hydrophilic characteristics. There are certain assumptions in the empirical formulas, so a large number of studies, along with sensitivity analysis, are needed during field validation for more accuracy in the estimation of evaporation. Due to resource and expertise limitations, Time-resolved interference could not be fully implemented to reach at the half-life period of the solutes over water. This method can be pursued for future investigations to assess evaporation rates using oil and water interference properly.

## Supplementary Information

Below is the link to the electronic supplementary material.

Supplementary file 1

## Data Availability

The dataset is available from the corresponding author on request.
