# Supplementary material for: Sustainable application of edible solute to control reservoir evaporation loss
Source: Sci Rep. 2025 Dec 24;15:44550. doi: 10.1038/s41598-025-28224-x (PMC12738850; doi:10.1038/s41598-025-28224-x)
Supplement: Supplementary file 1 [file 41598_2025_28224_MOESM1_ESM.docx]

**Table S1.** Assumptions and limitations of various empirical methods for evaporation estimation

| **Sr. No.** | **Equations used for Empirical Methods** | **Assumption** | **Limitation** |
| --- | --- | --- | --- |
| 1. | *Meyer’s Formula*  $\boldsymbol{E}_{\boldsymbol{L}}\boldsymbol{=}\boldsymbol{K}_{\boldsymbol{M}}\left( \boldsymbol{e}_{\boldsymbol{w}\boldsymbol{-}}\boldsymbol{e}_{\boldsymbol{a}} \right)\left[ \boldsymbol{1}\boldsymbol{+}\frac{\boldsymbol{u}_{\boldsymbol{9}}}{\boldsymbol{16}} \right]$ | Evaporation is proportional to the vapour pressure difference between the water surface and the air.  • Wind function is linear and measured at 9 m height.  • Applies to open water surfaces under steady climatic conditions. | • Valid only for lakes and reservoirs; not suitable for soil or cropped surfaces.  • Neglects the influence of solar radiation and heat storage.  • Errors occur under extreme climatic conditions and varying wind heights. |
| 2. | Rohwer’s Formula  $\boldsymbol{E}_{\boldsymbol{L}}\boldsymbol{=}\boldsymbol{0}\boldsymbol{.}\boldsymbol{771}\boldsymbol{(}\boldsymbol{1}\boldsymbol{.}\boldsymbol{465}\boldsymbol{-}\boldsymbol{0}\boldsymbol{.}\boldsymbol{000732}\boldsymbol{p}_{\boldsymbol{a}}\boldsymbol{)(}\boldsymbol{0}\boldsymbol{.}\boldsymbol{44}\boldsymbol{+}\boldsymbol{0}\boldsymbol{.}\boldsymbol{0733}\boldsymbol{u}_{\boldsymbol{0}}{\boldsymbol{)(}\boldsymbol{e}}_{\boldsymbol{w}}{\boldsymbol{-}\boldsymbol{e}}_{\boldsymbol{a}}\boldsymbol{)}$ | • Evaporation depends on vapour pressure deficit, wind speed near the surface, and atmospheric pressure.  • Atmospheric pressure correction is linear.  • Assumes uniform temperature distribution over the water surface. | Applicability limited to open water bodies; unsuitable for land/crop ET.  • Empirical constants may vary with climatic region.  • Solar radiation and cloud effects are not explicitly considered.  • Accuracy decreases at high altitudes and under variable climates. |
| 3. | Penman equation  $\boldsymbol{E}_{\boldsymbol{0}}\boldsymbol{=}\frac{\boldsymbol{(}\boldsymbol{700}\boldsymbol{T}_{\boldsymbol{M}}\boldsymbol{)/}\left( \boldsymbol{100}\boldsymbol{-}\boldsymbol{A} \right)\boldsymbol{+}\boldsymbol{15}\left( \boldsymbol{T}\boldsymbol{-}\boldsymbol{T}_{\boldsymbol{d}} \right)}{\left( \boldsymbol{80}\boldsymbol{-}\boldsymbol{T} \right)}$ | Evaporation is a function of temperature and vapour pressure deficit.  • Air temperature is the dominant climatic variable.  • Assumes uniform reference crop or water surface. | Applicability limited to open water bodies; unsuitable for land/crop ET.  • Empirical constants may vary with climatic region.  • Solar radiation and cloud effects are not explicitly considered.  • Accuracy decreases at high altitudes and under variable climates. |
| 4. | Blaney Criddle Equation  $\boldsymbol{E}_{\boldsymbol{L}}$=$\left( \boldsymbol{0}\boldsymbol{.}\boldsymbol{0173}\boldsymbol{T}_{\boldsymbol{a}\boldsymbol{-}}\boldsymbol{0}\boldsymbol{.}\boldsymbol{314} \right)\boldsymbol{T}_{\boldsymbol{a}}\frac{\boldsymbol{D}}{\boldsymbol{D}_{\boldsymbol{TA}}}$25.4 | Evaporation depends on air temperature and vapour pressure deficit ratio.  • Radiation and wind effects are assumed to be indirectly included in T and D terms.  • Constants derived from empirical calibration. | Wind, radiation and local heat storage are not explicitly considered.  • Constants vary with climatic zone and require regional calibration.  • Not reliable in extreme humid/arid conditions.  • Valid only for open water evaporation. |
